# Spontaneous droplets gyrating via asymmetric self-splitting on heterogeneous surfaces

**DOI:** 10.1038/s41467-019-08919-2

**Published:** 2019-03-05

**Authors:** Huizeng Li, Wei Fang, Yanan Li, Qiang Yang, Mingzhu Li, Qunyang Li, Xi-Qiao Feng, Yanlin Song

**Affiliations:** 1 Key Laboratory of Green Printing, CAS Research/Education Center for Excellence in Molecular Sciences, Institute of Chemistry, Chinese Academy of Sciences, 100190 Beijing, P. R. China; 2 University of Chinese Academy of Sciences, 100049 Beijing, P. R. China; 3 AML, CNMM and Department of Engineering Mechanics, and State Key Laboratory of Tribology, Tsinghua University, 100084 Beijing, P. R. China

## Abstract

Droplet impacting and bouncing off solid surface plays a vital role in various biological/physiological processes and engineering applications. However, due to a lack of accurate control of force transmission, the maneuver of the droplet movement and energy conversion is rather primitive. Here we show that the translational motion of an impacting droplet can be converted to gyration, with a maximum rotational speed exceeding 7300 revolutions per minute, through heterogeneous surface wettability regulation. The gyration behavior is enabled by the synergetic effect of the asymmetric pinning forces originated from surface heterogeneity and the excess surface energy of the spreading droplet after impact. The findings open a promising avenue for delicate control of liquid motion as well as actuating of solids.

## Abstract (web summary)

Controlling droplet impact and rebound behaviour can have applications in inkjet printing and self-cleaning. Here the authors show how a chemically-patterned surface with high-adhesive spirals surrounded by hydrophobic, low-adhesive regions leads to gyration behaviour of impacting droplets.

## Introduction

Controlling droplet-solid impacting behaviors^1,2^ is significant in a wide range of applications including self-cleaning^3,4^, anti-icing^5,6^, and inkjet printing^7–9^. The outcomes of a droplet impact on solid surfaces, such as deposition, rebounding, splashing, depend on both the micro/nanostructures and chemical property of the solid. Diverse strategies have been exploited by nature as well as artificial materials to regulate the droplet impact processes as well as the subsequent droplet motions^10–13^. For example, various superhydrophobic surfaces are fabricated to accelerate the droplet bouncing off solids after impact^14–16^; topological heterogeneity is utilized for directional transportation of impacting droplets^17,18^. However, due to the deformability of the droplet and the milliseconds-scale interaction^19^ between the impacting droplet and the solid, it is still a challenge to elaborately manipulate the impacting behaviors.

As an important mechanical law, the “Newton’s Law of Impact” depicts the elasticity of two objects collision by considering the approach and recession velocities of the objects. Normally the recession of a ball is in a linear motion if it vertically impacts on a solid wall with translational kinetic energy. The principle is also applicable with the reported researches relating to droplet impact on solid surfaces, where the droplet bounces in a translational type after impacting on a flat surface^15,19,20^.

In this work, we report a droplet rotational bouncing by impacting it on an adhesion-patterned surface, which seems beyond the “*Newton’s Law of Impact*”. Based on the mechanics modeling, we reveal that the angular momentum of the droplet is formed by the asymmetric adhesion forces that accumulate during the liquid film retraction. Under a proper test design, the maximum droplet rotational speed can be larger than 7300 revolutions per minute (rpm). We further demonstrate that the heterogeneous solid–liquid interaction forces during droplet impact can be exploited to drive sophisticated motions of the solids as well as the droplets.

## Results

### Droplet rotational bouncing

**Fig. 1:**
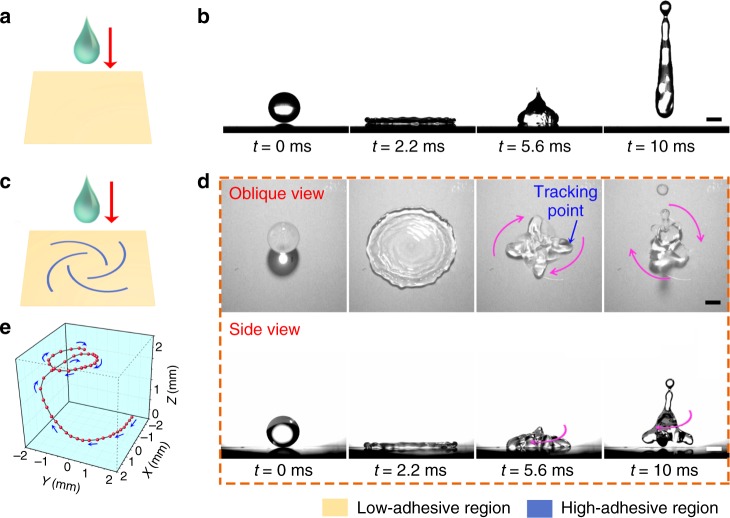
Droplet impact dynamics. **a**, **b** Scheme and sequenced images of a water droplet impacting on a homogeneous hydrophobic and low-adhesive surface. The droplet spreads, homogeneously recedes, and bounces vertically upward from the surface. **c** Scheme of a water droplet impacting on a low-adhesive substrate with high-adhesive patterns. **d** Selected snapshots to show the synchronized oblique and side views of the droplet gyrating by impacting on the deliberately-designed adhesion-patterned substrate. After reaching maximum spreading and forming a circular film, the liquid heterogeneously recedes accompanied by rotating in a pinwheel morphology. Four-lobed droplet gyrating in the air is achieved when the droplet departs from the substrate. **e** Three-dimensional trajectory of the tracking point marked at *t* *=* 5.6 ms in **d**. Time interval between the dots in the curve is 0.33 ms. In the tests above, the droplet diameter is 2.1 mm and the Weber number is 93. Scale bars: 1 mm

When a water droplet impacts on a hydrophobic and low-adhesive surface (Fig. 1a), it typically spreads into a liquid film at first^21^, and then uniformly recedes and rebounds from the substrate^22^ due to the water-repellency^23–25^, as shown in Fig. 1b and Supplementary Video 1. Although there are a handful of attempts, where the rebound direction can be regulated, the impacting and bouncing motions of the droplets are primarily translational. Here, by preparing a hydrophobic solid surface with specially designed chemical heterogeneity, we demonstrated that a droplet bouncing with gyration could be achieved. The chemically-patterned surface consists of high-adhesive spirals surrounded by hydrophobic and low-adhesive region (Fig. 1c and Supplementary Figure 1). As shown by the snapshots of synchronized oblique and side views in Fig. 1d, the droplet initially spreads into a circular film similar to that on the homogeneous low-adhesive substrate. However, a distinctive behavior occurs at *t* *=* 5.6 ms such that the droplet recedes non-axisymmetrically and forms a pinwheel-like morphology (see the hydrodynamics in Supplementary Figure 2). After the retraction proceeds to the center, the droplet rebounds from the substrate with a lobed gyrating behavior, as shown at *t* *=* 10 ms in Fig. 1d and Supplementary Video 2. To illustrate the spatial motion of the receding droplet in a more quantitative way, we tracked a material point in the droplet (as marked at *t* = 5.6 ms in Fig. 1d) and plotted its trajectory in Fig. 1e. The three-dimensional-spiral path clearly illustrates that the tracking point initially gyrates on the surface and then swirls up. This gyration motion is in sharp contrast to the previous reports involving drop impact^14,15,26^ or the collisions depicted in Newton’s Law of impact, where both the input and output movements are primarily translational.

**Fig. 2:**
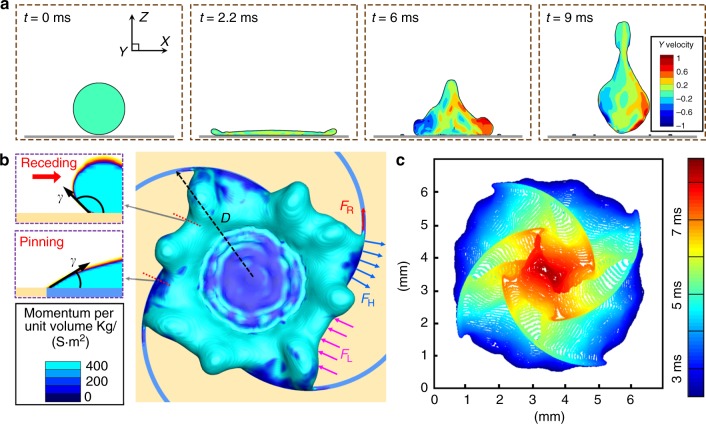
Mechanical analysis of the droplet gyration. **a** Simulated results to show the out-of-plane velocity distribution in the cross-section of the droplet at typical stages of before contact (*t* = 0 ms), maximum spreading (*t* = 2.2 ms), receding (*t* = 6 ms), and departing from the solid (*t* = 9 ms). **b** Force model to analyze the origin of the droplet gyration motion. A symmetric unit is used for the analysis due to its symmetry. *θ*_L_ and *θ*_H_ are the contact angles of the liquid film on the low-adhesive region and the high-adhesive spiral region, as shown by the simulated cross-sections. The driving forces from the solid to the liquid contain the force on the low-adhesive region (*F*_L_, *γ*cos*θ*_L_ per unit length, as marked by the pink arrows) and the force perpendicular to the high-adhesive spiral (*F*_H_, *γ*cos*θ*_H_ per unit length, as marked by the blue arrows). Meanwhile, the liquid film remained on the high-adhesive spiral brings resistance to the droplet rotating (*F*_R_, *γ* per unit length, as marked by the red arrows). *D* is the maximum spreading radius of the impacting droplet. The color shows the distribution of the momentum per unit volume. **c** Contours of the three-phase contact lines at different time of the retraction reveal that the liquid on the low-adhesive region recedes faster than that on high-adhesive spirals throughout the retraction process

To elucidate the physical origin of the gyrating motion, we performed numerical simulations of the impacting droplet using the coupled Level-Set and Volume of Fluid (CLSVOF) method (Supplementary Note 1 and Supplementary Video 3). The sequential images in Fig. 2a show the evolution of the droplet shape and the distribution of the out-of-plane velocity in the cross-sections of the droplet. During the spreading stage (from *t* = 0–2.2 ms), the translational kinetic energy is converted to the surface energy of the droplet as well as heat due to the viscous dissipation^27,28^. When the liquid film recedes, the out-of-plane velocity obviously enlarges antisymmetrically (*t* = 6 ms). On the left part, there is a large inward flow velocity, while on the right side the flow direction is opposite, indicating a clockwise gyrating movement. In this period, the surface energy is converted to both translational and rotational kinetic energy. The droplet gyrating persists during the receding process and even after the rebounding (*t* = 9 ms). It provides a facile strategy to convert the translational kinetic energy of impacting drops to prescribed rotational kinetic energy, which is crucial for energy harvesting or electricity generation^29^. Meanwhile, the rotational motion of liquid drops is also beneficial for phase mixing and material dispersion. As surface tension is analogous to other cohesive forces, a gyrating droplet can be used to mimic the shape evolution of biological/astronomical systems^30,31^.

As the droplet rotational behavior occurs during the retraction stage, we calculated the distribution of the magnitude of the momentum per unit volume at a typical moment (*t* *=* 4 ms) in Fig. 2b. The results suggest that the liquid on the hydrophobic and low-adhesive region retracts with a receding contact angle of *θ*_L_, while it is pinned by the hydrophilic and high-adhesive spirals with a contact angle of *θ*_H_, as illustrated in the insets of Fig. 2b. There exist net forces from the solid surface acting on the three-phase contact line (TCL): the force on the low-adhesive region (*F*_L_, $\gamma {\mathrm{cos}}\, \theta _{\mathrm{L}}$ per unit length, as marked by the pink arrows), and the force perpendicular to the high-adhesive spiral (*F*_H_, $\gamma {\mathrm{cos}}\, \theta _{\mathrm{H}}$ per unit length, as marked by the blue arrows)^32^. Meanwhile, the resistive force (*F*_R_) along with the spiral is simplified as the liquid surface tension (*γ*) per unit length, as marked by the red arrow. Based on the geometric calculations (Supplementary Note 2), the driving (*M*_D_, generated by *F*_L_ and *F*_H_) and resisting (*M*_R_, generated by *F*_R_) moments are approximated as $M_{\mathrm{D}} = \gamma ({\mathrm{cos}}\, \theta _{\mathrm{H}} - {\mathrm{cos}}\, \theta _{\mathrm{L}})\frac{{L_{\mathrm{i}}^{2} - L_{\mathrm{o}}^{2}}}{2}$ and $M_{\mathrm{R}} = \gamma W\frac{{R^{2} + L_{\mathrm{i}}^{2} - C^{2}}}{{2R}}$. Here *L*_i_ and *L*_o_ are the radii of the liquid at the inside and outside edges along the spiral; *R*, *W*, and *C* are the spiral radius, the spiral width and the center-to-center distance between the spiral and the pattern, respectively (schematics of these parameters are shown in Supplementary Figure 3). After proper simplification (Supplementary Note 2), the angular momentum (*T*) of the droplet at the end of the retraction stage is expressed as:

1 $$T \approx \frac{{N\gamma }}{6}\left( {\cos \theta _{\mathrm{H}} - \cos \theta _{\mathrm{L}}} \right)D^2\Delta \tau$$

where *D* is the radius of the droplet maximum spreading, Δτ is the time interval of the liquid receding along the inside and outside edges of the spiral. To testify that the droplet angular momentum originates from the unbalanced pinning forces distribution during the liquid retraction, the contours of the TCL at different time of the retraction are extracted and plotted in Fig. 2c. The contour plot clearly indicates that the liquid on the low-adhesive region always recedes faster than that on the high-adhesive spiral, suggesting the pinning force difference between the two regions is persistent throughout the retraction process.

**Fig. 3:**
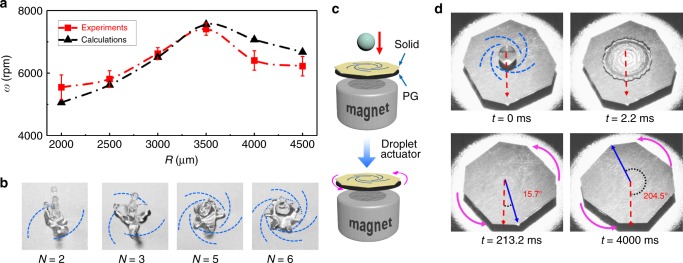
Droplet movement regulation and solid actuation. **a** Dependence of the instantaneous rotational speed of the droplet (*ω*) on the radius of the spiral (*R*). In these tests, the Weber number of the droplet is 93. **b** Various morphologies of the gyrating droplets by impacting on high-adhesive patterns with different spiral numbers *N* of 2, 3, 5, and 6, respectively. **c** Scheme of the droplet actuator where a chemically heterogeneous thin solid sitting on a magnetically-levitated pyrolytic graphite (PG) flake. Water droplet is released and impacts on the center of the solid. **d** Sequenced images to show a water droplet (4.8 mg in mass) impacting on the PG-supported heterogeneous solid (94.3 mg in total mass). The substrate is driven to rotate in a desirable direction for more than 8000 ms. The Weber number of the droplet is 93. The thicknesses of the PG flake and the patterned solid are 0.5 mm and 50 μm, respectively. In **b** and **d**, the blue dashed arcs represent the high-adhesive pattern on the low-adhesive surface

Because the gyration actuation critically relies on the asymmetric pinning forces induced by surface heterogeneity, it can be regulated by the spiral pattern. To investigate the quantitative dependence, we performed a series of impact tests by varying the radii of the high-adhesive spirals and recorded the droplet rotational speed (Supplementary Figure 4). As summarized in Fig. 3a, with the spiral radius enlarging from 2000 to 4500 μm, the droplet rotational speed initially increases to a maximum of about 7300 rpm when 3500-μm-spiral is adopted, then gradually decreases at larger radii. The correlation can be explained by the mechanics model performed in Fig. 2b and Supplementary Note 3. Based on the calculation, the theoretical droplet rotational speed at different sized spirals is illustrated by the dashed black line in Fig. 3a. The agreement between the calculation and the experimental data suggests that the mechanics model well captures the essentials of the droplet gyrating process. Meanwhile, altering the spiral number directly affects the morphology of the gyrating droplet and various lobed gyrating droplets can be obtained, as shown in Fig. 3b and Supplementary Video 4. In addition, the generality of this gyration actuation phenomenon is further demonstrated using various liquids on diverse solids (Supplementary Figure 5).

### Solid rotation actuated by impacting droplets

Since the pinning forces can provide moments to the liquid droplet^33^, the interaction forces should also exert a torque on the solid surface, which may be used for actuating the substrate. To test this idea, we placed a thin solid sheet with a similar heterogeneous surface pattern on a magnetically-levitated pyrolytic graphite (PG) flake. Then a water droplet is released to impact on the center of the solid (Fig. 3c). Due to the strong diamagnetic effect of PG^34,35^, the flake remained suspended with minimal friction during the whole impacting process (Supplementary Video 5). When the droplet impacted on the solid surface, it was regulated by the surface heterogeneity and retracted with rotation after reaching the maximum spreading. Meanwhile, the asymmetric solid–liquid interaction forces also caused the PG flake to rotate in the opposite direction (Fig. 3d), of which the rotational energy can be collected and utilized for hydroelectric power generation. The rotation speed of the PG flake could reach about 50° per second before it eventually stopped due to the eddy current and air damping (Supplementary Figure 6 and Supplementary Video 6). Considering that the weight of the solid and the PG flake (94.3 mg in total) is 20 times of the droplet (4.8 mg), the actuation capability from the heterogeneous interfacial forces is rather striking.

### General principle for droplet bouncing regulations

**Fig. 4:**
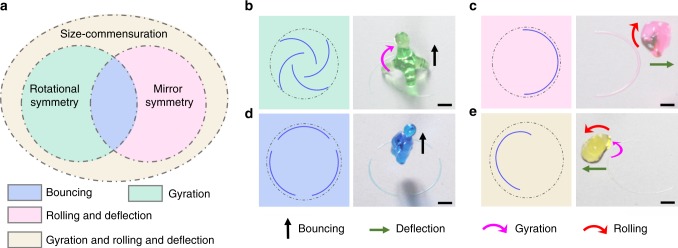
Exploitation of pinning forces for various droplet actuation. **a** Effect of the size-commensuration, rotational symmetry, and mirror symmetry of the adhesion pattern under the droplet maximum spreading on the impacting behaviors. **b** The droplet upward bounces with gyration after impacting on a low-adhesive surface with high-adhesive arcs that has rotational symmetry but no mirror symmetry. **c** A droplet deflects and rolls after impacting on a low-adhesive surface with a high-adhesive arc that is mirror symmetric but not rotationally symmetric. **d** The droplet bounces vertically upward after impacting on a low-adhesive surface with high-adhesive circular segments that are rotationally and mirror symmetric. **e** A droplet gyrates, rolls, and deflects after impacting on a low-adhesive surface with a high-adhesive Archimedean spiral segment that is asymmetric. The schemes of the patterns are given in the first column of **b**–**e**, in which the dashed circles represent the maximum spreading boundaries of the droplets. The scale bars are 1 mm

In addition to gyration motion, the pinning forces can be further exploited to achieve sophisticated droplet actuation (Supplementary Video 7). As the asymmetric pinning forces used for actuating droplets are induced by surface heterogeneity, the size of the adhesion pattern needs to be coupling with the droplet (Supplementary Figure 7). When the pattern size is commensurate with that of the droplet, different actuation can be achieved depending on the symmetry of the pattern under the maximum spreading of the droplet. Figure 4a illustrates the pattern design diagram. For a droplet impacting on a patterned surface with mirror symmetry, the net horizontal moment exerted by the solid to the liquid is zero, meaning that gyration would not occur. Similarly, a patterned surface with rotational symmetry corresponds to a zero net horizontal force and the droplet upward bounces. Rationally incorporating the symmetries of the adhesion pattern can induce diverse droplet actuations after impact. For example, when the pattern is rotationally symmetric but not mirror symmetric, the droplet can gyrate clockwise or anticlockwise depending on the chirality of the pattern after impacting (Fig. 4b and Supplementary Figure 8). By contrast, the droplet rolls and deflects after impacting if the pattern has mirror symmetry but no rotational symmetry (Fig. 4c). If the droplet impacts on a patterned surface that meets both symmetries, it would bounce vertically upward as indicated in Fig. 4d. Finally, coupled droplet behaviors, including deflection, gyration, and rolling, occur simultaneously when the adhesion pattern is asymmetric (Fig. 4e and Supplementary Figure 9).

## Discussion

In conclusion, we have achieved a distinctive droplet gyration induced by the asymmetric pinning forces arising from the solid–liquid interaction when a water droplet impacts a chemically heterogeneous substrate. More importantly, we demonstrated that exploiting the pinning forces can be a general strategy for attaining sophisticated droplet motions, which opens an avenue in future explorations, such as matter transportation^36^, energy transformation^37^, and object actuation^38^.

## Methods

### Fabrication of adhesion-patterned substrates

Porous alumina plates (Beijing NanoThink Printing Co., Ltd., China) are sequentially washed with ethanol, acetone, and deionized water, then blow-dried with nitrogen and surface-modified with 1H, 1H, 2H, 2H-perfluorodecyltrimethoxysilane (PFOTS) by chemical vapor deposition (CVD) at 80 °C for 2 h. The obtained alumina plates are low adhesive and hydrophobic. The substrates are covered with a photomask and exposed under a UV light (400 W, 365 nm) for 8 h. The exposed regions turn superhydrophilic (high-adhesive). Scanning electron microscope (SEM), static and receding contact angles characterization of the substrate are shown in Supplementary Figure 1.

For the substrate used for droplet actuator (Fig. 3d), thin Al plates (50 μm in thickness) are sequentially washed with ethanol, acetone, and deionized water, then immersed into Beck etchant for 10 seconds at room temperature. Beck etchant is prepared by mixing 40 mL HCl (37 wt %), 12.5 mL H_2_O and 2.5 mL HF (40 wt %) in a plastic bottle. The plates are blow-dried with nitrogen and surface-modified with PFOTS by CVD at 80 °C for 2 h. The obtained Al plates are superhydrophobic with ultra-low adhesion. The substrates are covered with a photomask and exposed to a UV light (400 W, 365 nm) for 8 h. The exposed regions turn superhydrophilic. SEM, static and receding contact angles characterization of the substrate are shown in Supplementary Figure 10.

### Drop impact process

The liquid droplet is formed by slowly squeezing the nozzle until the droplet detaches under gravity, with an initial speed of zero. Impact speed is adjusted by regulating the height of the nozzle. The diameter of the water droplets is 2.1 mm. The impact behaviors are recorded using high-speed cameras: Phantom V12.1 and Phantom VEO401L (Vision Research Inc.).

### Characterization

Morphology of the substrate is investigated on an SEM (F7500, JEOL). Dynamic and static contact angles are measured on an optical contact angle measurement equipment (DSA100, Kruss Co. Germ.). Adhesion force is obtained with a highly sensitive micro-electromechanical balance system.

## Supplementary information

Supplementary Information

Description of Additional Supplementary Files

Supplementary Movie 1

Supplementary Movie 2

Supplementary Movie 3

Supplementary Movie 4

Supplementary Movie 5

Supplementary Movie 6

Supplementary Movie 7

## Data Availability

The data that support the findings of this study are available from the corresponding author on reasonable request.
