# Supplementary material for: Spontaneous droplets gyrating via asymmetric self-splitting on heterogeneous surfaces
Source: Nat Commun. 2019 Mar 5;10:950. doi: 10.1038/s41467-019-08919-2 (PMC6401179; doi:10.1038/s41467-019-08919-2)
Supplement: Supplementary file 1 — Supplementary Information [file 41467_2019_8919_MOESM1_ESM.pdf]

Supplementary Materials

Spontaneous droplets gyrating via asymmetric self-splitting on heterogeneous surfaces

Li et al.

Contents

Supplementary Figures 1-10

Supplementary Notes 1-3

Supplementary References

Supplementary Figures

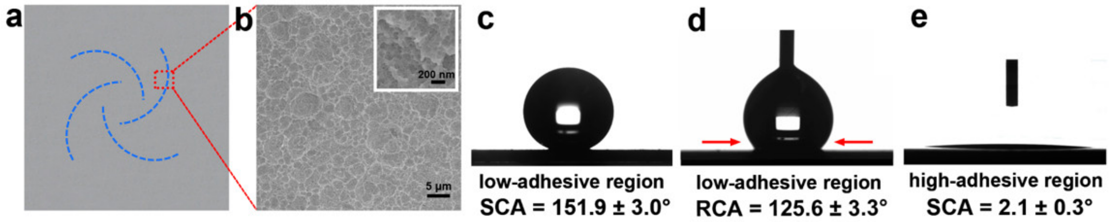

Supplementary Figure 1 | Morphology, structure, static and receding contact angles characterization of the heterogeneous substrate. a, Optical image of the substrate. The blue spirals indicate the high-adhesive pattern. The substrate is physically homogeneous and chemically heterogeneous. **b**, SEM images in different magnification to show the micro/nanostructures of the substrate. **c, d**, Static and receding contact angles of the low-adhesive region, indicating its water-repellency. **e**, Static contact angle of the spiral, showing its high adhesion.

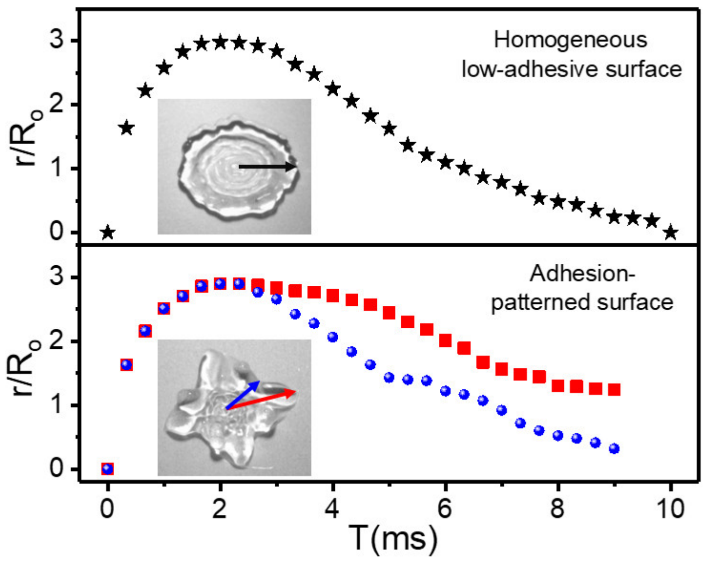

Supplementary Figure 2 | Droplet impact dynamic comparison between the homogeneous low-adhesive surface and the adhesion patterned surface. On the homogeneous surface, the droplet spreads and reaches its maximum spreading with a spreading ratio (r/R_o) of 3 at t = 2.2 ms. Then the liquid film uniformly recedes and after 10 ms the droplet departs from the surface. On the heterogeneous surface, the droplet uniformly spreads and reaches its maximum spreading into a circular liquid film with a spreading ratio (r/R_o) of 3 at t = 2.2 ms, which is the same as that on the homogeneous surface. However, in the receding process, the spreading ratio of the liquid at the outside edge of the spiral (indicated by the red arrow) decreases slower than that at the inside edge of the spiral (indicated by the blue arrow). After 9 ms, the gyrating droplet bounces off the surface.

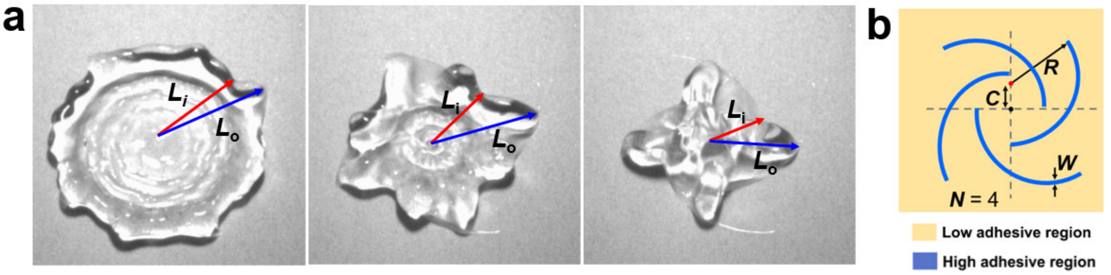

Supplementary Figure 3 | Parameter schematics. a, Sequenced images to show the changing of the liquid radii at the inside edge (L_i) and outside edge (L_o) of the spiral. **b**, Parameters describing the adhesion pattern, including the spiral number (N), the spiral width (W), the spiral radius (R) and the center-to-center distance between the spiral and the pattern (C).

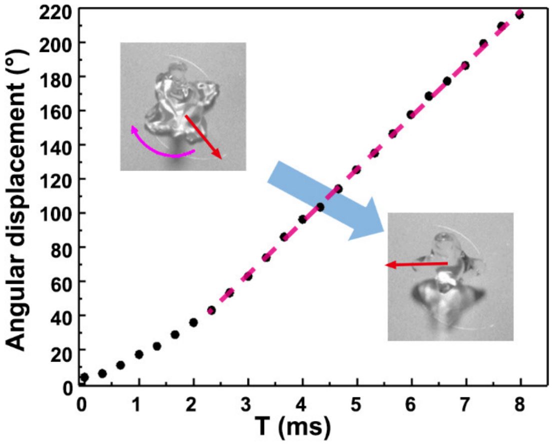

Supplementary Figure 4 | Angular displacement (*φ*) as a function of time (*t*) for a four-lobed gyrating droplet. Insets are the typical behaviors in the droplet gyrating. The shape of the water droplet gradually becomes a liquid ball under the effect of the surface tension, so the receding process and the initial stage after bouncing from the surface are investigated, where the droplet has obvious lobed morphology. The angular velocity, which is the slope of this curve, initially increases and then remains unchanged during the investigated period. So the angular velocity V is calculated by $V = \frac{\Delta\varphi}{\Delta t}$. Rotational speed (rpm) is calculated by $\omega = \frac{60V}{2\pi} = \frac{30V}{\pi}$. In this case, $V = \frac{\Delta\varphi}{\Delta t} = 605$ rad/s, $\omega = \frac{30V}{\pi} = 5787$ rpm.

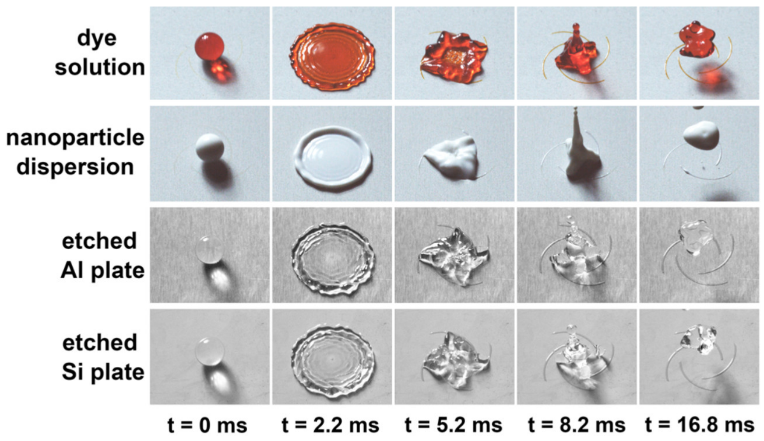

Supplementary Figure 5 | Generality demonstration of the droplet gyration. From up to bottom: a droplet of methyl orange solution impacting on a chemically heterogeneous porous alumina plate, a droplet of polystyrene nanoparticles dispersion impacting on a chemically heterogeneous porous alumina sheets, a droplet of water impacting on a chemically heterogeneous etched-Al plate, and a droplet of water impacting on a chemically heterogeneous etched-Si plate. The size of the droplets is 2.1 mm in diameter, and the Weber number is 93 (1.8 m/s).

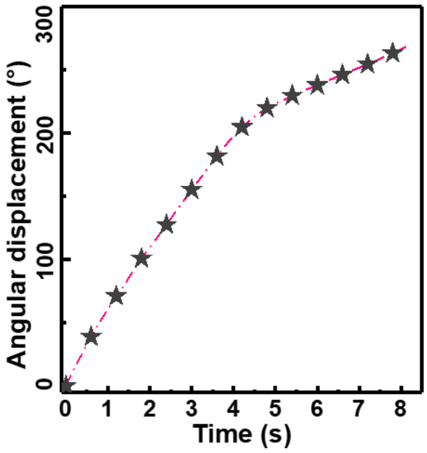

Supplementary Figure 6 | Angular displacement variation of the solid actuated by a gyrating droplet. In the initial stage (0-4 ms), the rotational speed (ω) of the solid calculated by the slope of the curve is 50.4 °/s.

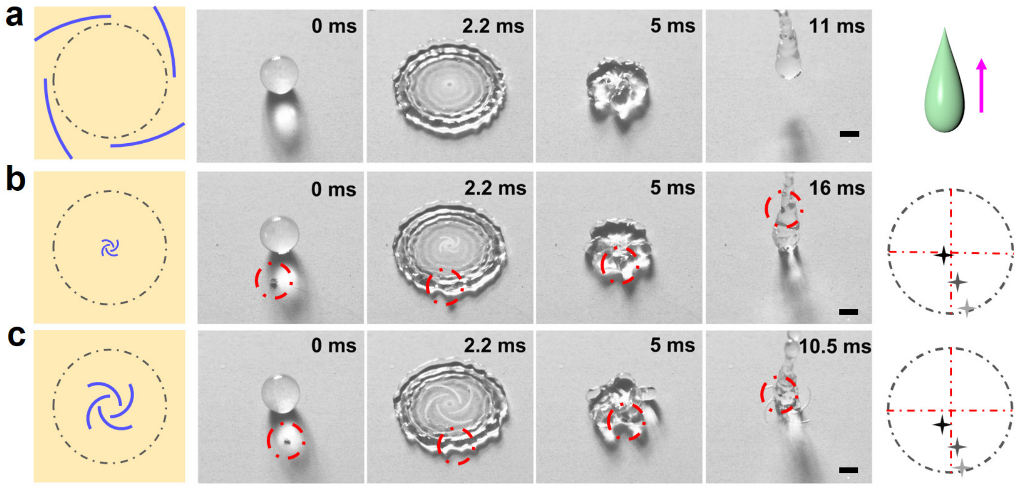

Supplementary Figure 7 | Droplet gyrating by impacting on incommensurate high-adhesive patterns. The schemes of the patterns are given in the first column. The dashed circles are the maximum spreading boundary of the droplet. **a**, The high-adhesive pattern is larger than the maximum spreading of the droplet. The droplet homogeneous spreads and receding, and finally upward rebounds from the substrate, as shown by the scheme in the last column. **b, c** The size of the patterns in **b** and **c** are 10 % and 50 % of that in Fig. 1d. To show the gyrating behaviors of the droplets, paper slices are placed on the solid surface before impacting. The slices are wetted and taken to recede by the liquid film. Horizontal projections of the paper slices are shown in the last column. The eccentric trajectories reveal that the droplets slightly rotate during the retraction. The scale bars are 1 mm.

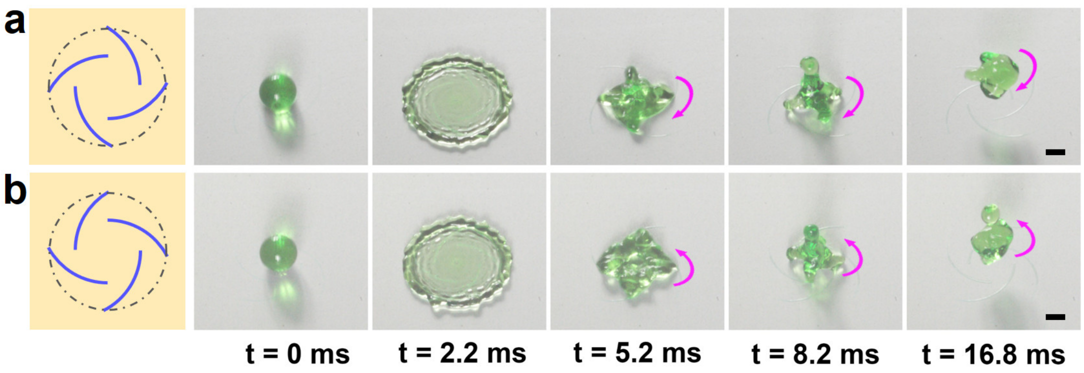

Supplementary Figure 8 | Clockwise and anticlockwise droplet gyration. The schemes of the patterns are given in the first column. The dashed circles are the maximum spreading boundary of the droplet. **a**, The droplet gyrates clockwise by impacting on a dextrorotary high-adhesive pattern. **b**, The droplet gyrates anticlockwise by impacting on a levorotary high-adhesive pattern. The scale bars are 1 mm.

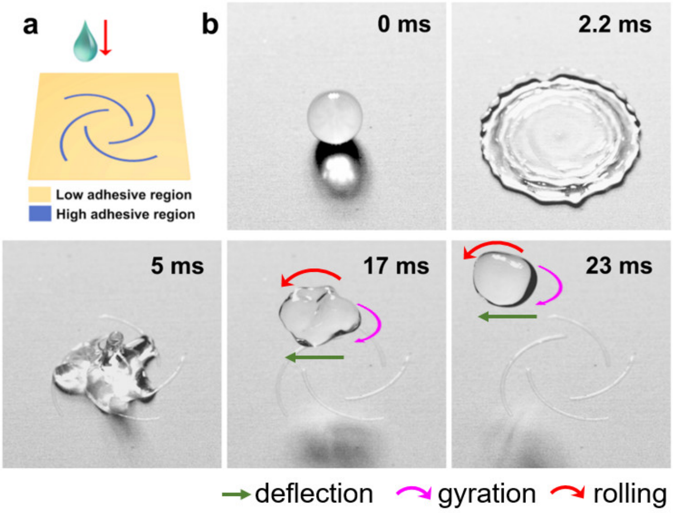

Supplementary Figure 9 | Coupled droplet actuation behaviors induced asymmetric adhesion-patterns. a, The schemes of the droplet and the adhesion-pattern that are not aligned. **b**, Sequenced snapshots to show the complex droplet bouncing behaviors including gyrating, rolling and deflection.

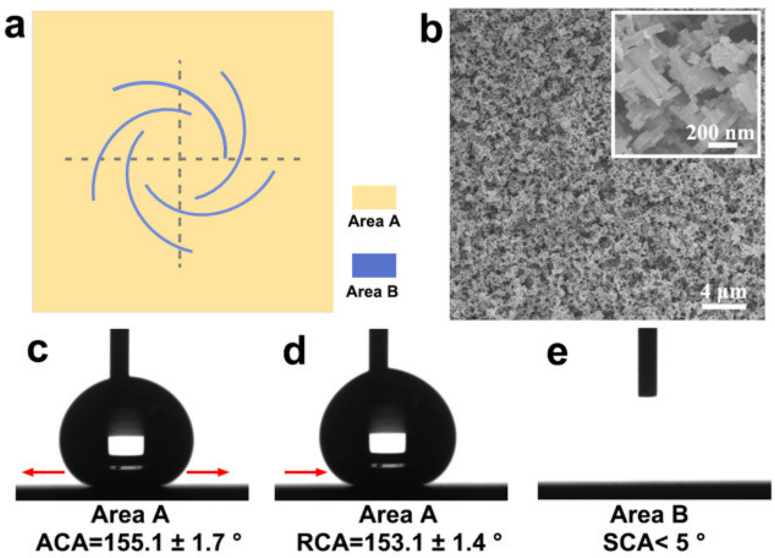

Supplementary Figure 10 | Scheme and characterization of the patterned substrate used for droplet actuator. a, Scheme of the pattern design, where the yellow color represents the superhydrophobic region, while the blue color represents the superhydrophilic region. The spiral width is 100 µm, the center-to-center distance is 1250 µm, and the spiral radius is 2500 µm, and the spiral number is 5. **b**, SEM images to show the micro-/nanostructure of the substrate. **c, d**, Advancing and receding contact angle (ACA and RCA) of the superhydrophobic region, indicating its low lateral-adhesion force. **e**, Static contact angle (SCA) of the superhydrophilic region.

Supplementary Notes

Supplementary Note 1: Numerical simulation of droplet gyrating using CLSVOF methods.

To have a deep understanding of droplet gyrating, the evolution process of an impact droplet on the chemically heterogeneous surface is modeled by the method of coupled Level-set and Volume of Fluid (CLSVOF) function[1-5].

The basic theory of CLSVOF function is introduced as follows. Let α_{p} denotes the volume fraction of the primary gas phase, and α_{s} the volume fraction of the secondary liquid phase. Since there are only two immiscible fluids in each cell, the sum of the volume fraction equals 1, that is

$$\alpha_{\mathrm{p}} + \alpha_{\mathrm{s}} = 1. \tag{1}$$

For the Volume of Fluid (VOF) method, the continuity equation of the secondary liquid phase can be expressed as

$$\frac{1}{\rho_{\mathrm{s}}}\left[\frac{\partial}{\partial t}\left(\alpha_{\mathrm{s}}\rho_{\mathrm{s}}\right) + \nabla\cdot\left(\alpha_{\mathrm{s}}\rho_{\mathrm{s}}\vec{v}_{\mathrm{s}}\right)\right] = 0. \tag{2}$$

where ρ_{s} and $\vec{v}_{\mathrm{s}}$ are the density and the velocity of the secondary phase, respectively. Due to the discontinuity of α_{s} across the interface, the VOF method has its drawbacks to calculate its spatial derivatives. Then a coupled Level-set function and VOF approach is employed to overcome this deficiency. With its spatial gradient calculated accurately, the Level-set function is smooth and continuous, defined as

$$\varphi(\vec{x},t) = \begin{cases} +|d| & \vec{x} \in \text{the primary phase} \\ 0 & \vec{x} \in \text{zero level set (interface)} \\ -|d| & \vec{x} \in \text{the secondary phase} \end{cases} \tag{3}$$

where d is the distance from a given point $\vec{x}$ in the domain to the liquid-gas interface at time t. And the conservation equation for the Level-set function φ reads

$$\frac{\partial\varphi}{\partial t} + \nabla\cdot(\vec{v}\varphi) = 0 \tag{4}$$

where $\vec{v}$ is the underlying velocity field. At each time step, φ must be reinitialized coupling with the VOF method to guarantee the conservation of mass. Based on the

Level-set function, the unit normal vector $\vec{n}$ of the liquid-gas interface can be estimated as

$$\vec{n} = \frac{\nabla \varphi}{|\nabla \varphi|}|_{\varphi=0} \tag{5}$$

In addition, when the interface is near a solid wall with a specified contact angle θ_w, $\vec{n}$ should be modified in the cells next to the wall that contain the interface by [6,7]

$$\vec{n} = \vec{n}_\mathrm{w} \cos\theta_\mathrm{w} + \vec{t}_\mathrm{w} \sin\theta_\mathrm{w} \tag{6}$$

where $\vec{n}_\mathrm{w}$ and $\vec{t}_\mathrm{w}$ are the unit normal and tangential vectors of the wall, respectively. Once given $\vec{n}$, the curvature κ of the interface is

$$\kappa = \nabla \cdot \vec{n} \tag{7}$$

Considering the effects of the surface tension and wall adhesion, the modified momentum equation is given as

$$\frac{\partial(\rho\vec{v})}{\partial t} + \nabla\cdot(\rho\vec{v}\vec{v}) = -\nabla p + \nabla\cdot\mu\left[\nabla\vec{v} + (\nabla\vec{v})^\mathrm{T}\right] + \rho\vec{g} + \vec{F} \tag{8}$$

where ρ is the mean density, p the pressure, μ the mean viscosity, $\vec{g}$ the gravitational acceleration, and $\delta(\varphi)$ the piecewise function. More details can be found in the reference[6].

Particularly, $\vec{F}$ is the momentum source item concerned with the tension σ and the curvature κ of liquid-gas interface. According to the continuum surface force (CSF) model[7], $\vec{F}$ can be expressed as

$$\vec{F} = \sigma\cdot\kappa\cdot\delta(\varphi)\nabla\varphi \tag{9}$$

Note that κ should be amended in connection with θ_w near the wall. Thereby, the moving process of liquid-gas two-phase flow interface can be well tracked and mass is conserved by solving the above equations.

Using the software ANSYS Fluent, the dynamic behavior of droplet gyrating is simulated with the method of CLSVOF function. Considering the asymmetry of the

solid wall, the computational domain with three-dimensional (3D) grids is a cylinder, charactered by a radius of 6 mm and a height of 6 mm, and it contains 5766600 hexahedral cells generated by swept method after the grid independence test. To improve the accuracy of the calculation, the near-wall grid is densified self-adaptively. The corresponding time step, 1.0e-6 s, is of good efficiency and convergence. The heterogeneous solid surface, defined as the non-slipping boundary, is divided into two parts: the low-adhesive region with a dynamic contact angle of 160° and the high-adhesive region with a contact angle of 10°. The geometric parameters of both regions are the same as the pattern design in Fig. 1d. The other boundaries are the conditions of pressure-outlet. The coupling equations of pressure and velocities were solved via the PISO (Pressure Implicit Split Operator) method. As for the two phases, the primary one is set as air, while the secondary one set as water. The water droplet, modeled as a 2.1-mm-diameter sphere with a downward initial velocity of 1.8 m/s and gravitational acceleration of 9.8 m/s^2, is just placed on the center of the solid surface. The simulated result is in good agreement with the experimental data, as shown in Video 3.

Supplementary Note 2: Mechanical analysis of the droplet gyration movement.

We adopt a typical liquid morphology in the receding process for the analysis.

The liquid on the hydrophobic and low-adhesive region retracts with a receding contact angle of θ_L. The force (F_L) from the low-adhesive region to the liquid film is $\gamma\cos\theta_L$ per unit length, and its direction is perpendicular to the receding liquid film (Supplementary Figure S11). The moment (M_L) of this force is calculated by:

$$M_L = \gamma \cos\theta_L S (L_i \cos\varphi - \frac{S}{2}) \quad 10)$$

where γ is the liquid surface tension, L_i is the radius of the droplet contact line at the inside edge of the high-adhesive arc, S is the length of the receding liquid on the low-adhesive region.

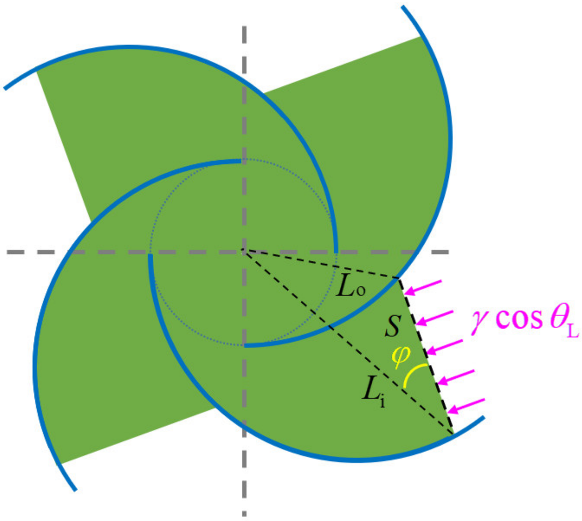

Supplementary Figure 11. Mechanical analysis on the hydrophobic and low-adhesive region.

The liquid is blocked to recede perpendicular to the high-adhesive arc with a contact angle of θ_H. The force (F_H) from the solid to the liquid is $\gamma\cos\theta_H$ per unit length, and its direction is perpendicular to the high-adhesive arc (Supplementary Figure S11). The moment (M_H) of the force is calculated as:

$$M_H=\int_{\beta_o}^{\beta_i}\gamma\cos\theta_H RC\sin\beta d\beta \quad (11)$$

where R is the radius of the arc, C is the center-to-center distance between the arc and the pattern, β is the angle of the arc in the azimuthal direction, and β_i *and* β_o are the angles where the liquid is still contacting the inside and outside edges of the arc.

The liquid is split on the high-adhesive arc and it brings resistance to the droplet rotating. The resistance force (F_R) along with the arc is simplified as the liquid-gas surface tension (γ) per unit length (Supplementary Figure S12). The moment (M_R) of the resistance force is calculated as:

$$M_R=\gamma WL_i\cos\alpha \quad (12)$$

where W is the width of the arc, α is the angle between the liquid radii of the pattern and the arc.

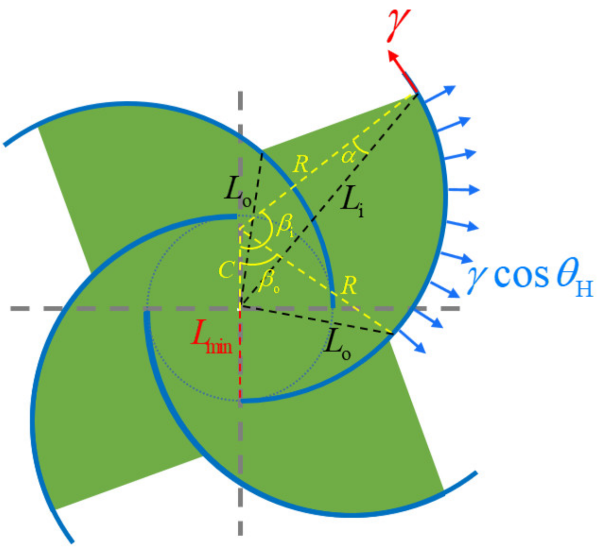

Supplementary Figure S12. Mechanical analysis on the high-adhesive arc.

We make the following assumptions to calculate the angular momentum of the liquid film at the end of the retraction: (1) θ_L and θ_H keep constant in the receding process; (2) L_i and L_o respectively decrease with constant velocities of v_i and v_o (L_o is the radius of the droplet contact line at the outside edge of the high-adhesive arc); (3) we set t = 0 ms when the liquid film begins to recede.

According to the assumptions, L_o and L_i are described as:

$$\begin{aligned} L_o &= D - v_o t,\ 0 < t < \tau_o \\ L_i &= L_{min} - v_i t,\ 0 < t < \tau_i \end{aligned} \tag{13}$$

where D is the maximum spreading radius of the impacting droplet, L_{min} is the distance between the pattern center and the nearest end of the high-adhesive arc, τ_o and τ_i are the total time needed for the liquid receding along the outside and inside edges of the high-adhesive arc and are determined experimentally. τ_o and τ_i are greatly influenced by the spiral parameters. We have:

$$v_o = \frac{D - L_{min}}{\tau_o},\ v_i = \frac{D - L_{min}}{\tau_i} \tag{14}$$

The liquid at the outside edge of the arc completes the retraction earlier than that at the inside edge ($\tau_o < \tau_i$), and we assume $L_o = L_{min}$ during the time interval between the

completion of the liquid retraction at the inside and outside edges of the arc ($\Delta\tau = \tau_i - \tau_o$).

The driving moment (M_D) of the liquid consists of M_L and M_H that are generated by F_L and F_H. So the total driving angular momentum (T_D) of the liquid film at the end of the retraction is calculated as:

$$\begin{aligned} T_D &= N\int M_D \mathrm{d}t = N\int_0^{\tau}\left[\int_{\beta_o}^{\beta_i} \gamma\cos\theta_H RC\sin\beta \mathrm{d}\beta - \gamma\cos\theta_L S(L_i\cos\varphi - \frac{S}{2})\right]\mathrm{d}t \\ &= \frac{N\gamma(\cos\theta_H - \cos\theta_L)(D^2 + DL_{min} - 2L_{min}^2)}{6}\Delta\tau \end{aligned} \quad (15)$$

where $\Delta\tau = \tau_i - \tau_o$. The moment of resistance (M_R) is induced by F_R. The resistance angular momentum (T_R) of the liquid film at the end of the retraction is calculated as:

$$\begin{aligned} T_R &= N\int M_R \mathrm{d}t = N\int_0^{\tau} \gamma W L_i \cos\alpha \mathrm{d}t = N\int_0^{\tau} \gamma W L_i \frac{R^2 + L_i^2 - C^2}{2RL_i}\mathrm{d}t \\ &= N\gamma W\left(\frac{D^2 + DL_{min} - 2L_{min}^2}{6R} + L_{min}\right)\tau_i \end{aligned} \quad (16)$$

So the total angular momentum (T) of the liquid is expressed as:

$$T = T_D - T_R \quad (17)$$

We note that T_R is much smaller than T_D and can be omitted when $(\cos\theta_H - \cos\theta_L) \gg \frac{W}{R}$. For the tests that the maximum spreading radius of the impacting droplet is approximately twice of L_{min}, the angular momentum (T) of the liquid at the end of retraction is expressed as:

$$T \approx \frac{N\gamma}{6}(\cos\theta_H - \cos\theta_L)D^2\Delta\tau \quad (18)$$

Supplementary Note 3: Detailed calculation of the droplet rotational speed.

For the droplet rotational speed calculation of the case in Fig. 3a, the spiral number (N) is 4, the liquid surface tension γ is 72.8 mN/m, θ_L and θ_H are 125° and 2.1°, respectively, the radius of the maximum spreading of the impacting droplet (D) is 3 mm, and the time intervals under different spiral radius (R) are:

R (μm)	2000	2500	3000	3500	4000	4500
τ_i (ms)	6.06	5.91	5.78	5.65	5.07	4.78
τ_o (ms)	4.00	3.62	3.13	2.57	2.17	2.15
$\tau_i - \tau_o$ (ms)	2.06	2.29	2.65	3.08	2.90	2.63

During the droplet gyrating in the air, the moment of inertia of the droplet is approximately expressed as $I = \frac{1}{2}mr^2$, where m is the mass of the droplet ($m \approx 4.8$ mg), r is the radius of the droplet (we take $r \approx 1.05$ mm for simplification). According to Equation (18), the calculated droplet rotational speed under different spiral radii are:

R (μm)	2000	2500	3000	3500	4000	4500
ω (rpm)	5056	5620	6504	7560	7069	6627

Supplementary References

1 Blake, J., Thompson, D., Raps, D. & Strobl, T. Simulating the freezing of supercooled water droplets impacting a cooled substrate. *Aiaa J* **53**, 1725-1739, (2015).
2 Shen, Y. Z. *et al.* Bouncing dynamics of impact droplets on the convex superhydrophobic surfaces. *Appl. Phys. Lett.* **110**, 221601, (2017).
3 Choi, M., Son, G. & Shim, W. A level-set method for droplet impact and penetration into a porous medium. *Comput. Fluids* **145**, 153-166, (2017).
4 Kagawa, Y. *et al.* Permeation of concentrated oil-in-water emulsions through a membrane pore: numerical simulation using a coupled level set and the volume-of-fluid method. *Soft Matter* **10**, 7985-7992, (2014).
5 Tanguy, S. & Berlemont, A. Application of a level set method for simulation of droplet collisions. *Int. J. Multiphase Flow* **31**, 1015-1035, (2005).
6 Fluent, A. Ansys fluent theory guide. *ANSYS Inc., USA* **15317**, 724-746, (2011).
7 Brackbill, J. U., Kothe, D. B. & Zemach, C. A continuum method for modeling surface-tension. *J. Comput. Phys.* **100**, 335-354, (1992).
