# Supplementary material for: Spontaneous droplets gyrating via asymmetric self-splitting on heterogeneous surfaces
Source: Nat Commun. 2019 Mar 5;10:950. doi: 10.1038/s41467-019-08919-2 (PMC6401179; doi:10.1038/s41467-019-08919-2)
Supplement: Supplementary file 2 — Description of Additional Supplementary Files [file 41467_2019_8919_MOESM2_ESM.pdf]

Video 1. Droplet impact and rebound on hydrophobic/low-adhesive surface. A water droplet impacts on a homogeneous hydrophobic/low-adhesive surface. The Weber number is 93. Static and receding contact angles of the substrate are 151° and 125°, respectively. The process is recorded at 15000 frames per second (fps) and shown at 60 fps.

Video 2. Four-lobed droplet gyration. A water droplet impacts on a chemically heterogeneous surface. The number, width, radius and center-to-center distance of the high-adhesive spirals are 4, 100 μm, 2500 μm, and 1250 μm, respectively. Static and receding contact angles of the hydrophobic and low-adhesive region are 151° and 125°, and the static contact angle of the high-adhesive spiral is 2.1° (superhydrophilic). The Weber number is 93. The process is recorded at 15000 fps and shown at 60 fps.

Video 3. Comparison of the droplet gyration with the simulated results. The left part is the droplet impact and gyrating process on a chemically heterogeneous surface. The Weber number is 93. The process is recorded at 15000 fps and shown at 20 fps. The right part is the simulated results of this process. The simulation parameters are the same as the experiments.

Video 4. Gyrating droplets with various morphologies. The number of the spirals ranges from 2 to 6. The width of the spirals is 100 μm, the center-to-center distances is 1250 μm, and the radius of the spirals is 2500 μm. The Weber number is 93. These processes are recorded at 15000 fps and shown at 100 fps.

Video 5. Droplet impact on a superhydrophobic solid floated by a magnetic suspension system. A superhydrophobic thin Al sheet is placed on a pyrolytic graphite (PG) flake that is suspended by the diamagnetic effect. A water droplet is controlled to impact on the center of the Al sheet, and the PG-supported sheet oscillates up and down due to the droplet impact. However, the PG flake has not contacted with the magnets during the whole process, indicating the large magnetic levitation capability of this system. The Weber number is 93. The process is recorded at 15000 fps and shown at 100 fps.

Video 6. Droplet actuator. A droplet impacts on a magnetically-levitated pyrolytic graphite (PG) flake (87.6 mg in mass) covered with a chemically heterogeneous solid (Al plate, 6.7 mg in mass). The width of the spirals is 100 μm, the center-to-center distance is 1250 μm, the radius of the spirals is 2500 μm, and the number of the spirals is 5. The Weber number is 93. The process is recorded at 5000 fps and shown at 100/5000 fps.

Video 7. Diversified droplet behaviors induced by surface heterogeneity. Water droplets with food colorings impact on hydrophobic and low-adhesive surfaces with hydrophilic and high-adhesive patterns. The movies correspond to the tests in Fig. 4 b-e. The Weber number is 93. The processes are recorded at 5000 fps and shown at 50 fps.
